# Introgression Breeding in Barley: Perspectives and Case Studies

**DOI:** 10.3389/fpls.2020.00761

**Published:** 2020-06-12

**Authors:** Javier Hernandez, Brigid Meints, Patrick Hayes

**Affiliations:** Department Crop and Soil Science, Oregon State University, Corvallis, OR, United States

**Keywords:** genetic resources, multi-rust resistance, haplotype, high throughput genotyping, genetic diversity, genetic mapping

## Abstract

Changing production scenarios resulting from unstable climatic conditions are challenging crop improvement efforts. A deeper and more practical understanding of plant genetic resources is necessary if these assets are to be used effectively in developing improved varieties. In general, current varieties and potential varieties have a narrow genetic base, making them prone to suffer the consequences of new and different abiotic and biotic stresses that can reduce crop yield and quality. The deployment of genomic technologies and sophisticated statistical analysis procedures has generated a dramatic change in the way we characterize and access genetic diversity in crop plants, including barley. Various mapping strategies can be used to identify the genetic variants that lead to target phenotypes and these variants can be assigned coordinates in reference genomes. In this way, new genes and/or new alleles at known loci present in wild ancestors, germplasm accessions, land races, and un-adapted introductions can be located and targeted for introgression. In principle, the introgression process can now be streamlined and linkage drag reduced. In this review, we present an overview of (1) past and current efforts to identify diversity that can be tapped to improve barley yield and quality, and (2) case studies of our efforts to introgress resistance to stripe and stem rust from un-adapted germplasm. We conclude with a description of a modified Nested Association Mapping (NAM) population strategy that we are implementing for the development of multi-use naked barley for organic systems and share perspectives on the use of genome editing in introgression breeding.

## Introduction

Introgression breeding has been an important method for improving barley since domestication, and it remains a key tool for expanding genetic diversity to meet current and future challenges to crop production. For the purposes of this chapter, we will define introgression as the transfer of one or several novel, favorable alleles from un-adapted germplasm to adapted germplasm. Barley serves as an excellent example for charting the history, current status, and future prospects for introgression breeding because it is a diploid genetic model for the Triticeae tribe and an important and versatile crop grown (nearly) from pole to pole. In our review, we will discuss the unique features of this crop, chart the evolution of tools for managing the introgression process, and look ahead to how introgression could be enhanced for both organic and conventional production systems.

## Domestication, Introgression and Current Status

The domestication of cultivated barley (*Hordeum vulgare* L) from *Hordeum vulgare subsp. spontaneum* Koch) began ∼10,000 years before present (Zohary and Hopf, 2000). Key domestication traits included determinate growth habit, increased seed set, greater inflorescence number, non-shattering, larger seed size, and more rapid germination (Harlan et al., 1973). These early domestication efforts surely involved a first step towards managed introgression. As early domesticators identified plants with novel phenotypes, they exchanged seeds of these plants with neighbors. Blended with existing seed stocks, the novel seeds would have led to the formation of heterogeneous mixtures of nearly homozygous lines (land races). Selection for novel phenotypes would increase their frequency in these land races, and the naturally occurring outcrossing (∼2%) that occurs in barley (Abdel-Ghani et al., 2004) and/or the environmentally induced outcrossing the can occur in selfing species (van Ginkel and Flipphi, 2020) would increase the frequency of favorable alleles introgressed into locally adapted genomes. Interestingly, many of the initial domestication traits remain critical in modern barley breeding.

The initial domestication of barley, based on archeological evidence, most likely occurred in the Fertile Crescent (Harlan and Zohary, 1966) with other possible sites in Central Asia and Africa (Morrell and Clegg, 2007; Dai et al., 2012). From these initial domestication sites, barley moved quickly into Europe and Asia, with mechanical mixtures and outcrossing facilitating a march estimated to have occurred at a pace of nearly 10 km/year (Morrell and Clegg, 2007). Ten thousand years after domestication began, barley is the fourth most widely grown cereal after wheat, maize, and rice and is planted in a wide range of environments around the world (Hayes et al., 1993; FAOSTAT, 2017).

Two phenotypes that today define the principal germplasm groups of cultivated barley were selected ∼8–9,000 years ago: inflorescence type (two-row vs six-row) and hull adherence type (covered vs naked). The fertility of the triad of florets at each rachis node determines the head type barley: two-row (ancestral) or six-row (selected post-domestication). In the former, only the central florets are fertile, whereas in six-row types, all three florets are fertile. The *Vrs1* gene responsible for head type was cloned by Komatsuda et al. (2007) and these authors described a single dominant allele and a number of loss-of-function six-row alleles that may have been selected at different places and times. The predominance of a particular allele/head type in a geographical region can, in some cases, be traced to which type of inflorescence was first introduced (introgressed) and certainly to end use. Two-row types are used predominately in the brewing and distilling industries because of the higher likelihood of uniform, plump kernels (Schwarz and Li, 2011). Because barley was, and is, used primarily for malting and distilling in Europe, the wild type, two-row allele predominates in the region. The six-row phenotype, in contrast, was selected and maintained in North Africa, the Iberian Peninsula, and eastern Asia, where brewing was not as prevalent. Both head types are present in regions where barley is used for feed and food. An interesting case study in introgression and head type is the rapid switch from six-row malting types to two-row malting types in North America that occurred in response to a re-direction of the malting and brewing industries in the 1990’s. Throughput the 20th century, the majority of North American malting barley was six-row due to perceived positive impact on beer flavor: today the American Malting Barley Association no longer supports research on six-row barley nor does it accept six-rows into its evaluation process (Craft Brewing Business, 2019). The rapid introgression of two-row spike morphology, in response to this shift, was accomplished thanks to targeted introgression of the *Vrs1* allele in two-row by six-row crosses and by the introduction of two-row varieties from Europe.

Today, most cultivated barleys are covered (hulled), meaning the lemma and palea adhere to the pericarp. Taketa et al. (2008) cloned the *Nud* gene, which is responsible for hull adherence. In *nud* genotypes, the seed threshes clean, as in wheat. The preferred botanical term for this phenotype is “naked,” although the term “hull-less” is also common. Covered types are preferred by the malting and brewing industries, because the hulls are used as natural filters during the brewing process (Newman and Newman, 2008). Barley varieties selected for feed production are also often covered, as selection for this end-use is based primarily on grain yield. It is important to note, however, that the hull accounts for ∼10% of the yield and is composed of insoluble fiber (Rey et al., 2009). Due to the higher economic value of malting barley compared to feed and food barley, in Europe and North American the focus of breeding efforts has been on agronomic and quality performance in covered two-row types destined for the malting and brewing industry (Newman and Newman, 2008; Meints et al., 2016). Naked barley is preferred for human consumption as hull removal requires additional processing, e.g., mechanical removal of the hull by “pearling” (Meints and Hayes, 2020). Naked barley is a staple food crop in the Himalayan region, the Andes, and the Ethiopian highlands. In Morocco, average consumption was recently reported at 28 kg/year (Aldughpassi et al., 2016). Barley is currently gaining popularity in western diets due to its health and nutritional benefits (Meints et al., 2016). Naked barley currently represents a small percentage of world barley production, as most barley is grown for feed and malt (Newman and Newman, 2008). However, systematic introgression of the naked phenotype is a goal of an ongoing collaborative breeding effort in North America to develop naked multi-use barleys for organic systems, as described later in this chapter.

### Introgression From Genetically Diverse Sources

With the rediscovery of Mendel’s work and the application of it to plant breeding, introgression in barley was made more systematic via controlled crossing. Specific examples from the early days of barley breeding are not obvious, most likely due to a focus on quantitative traits such as yield and resistance to some diseases. With a focus on improvements in yield and grain quality, a common breeding strategy was based on crossing elite by elite material. This led to an inevitable narrowing of the germplasm base (Bernardo, 2014) although, interestingly, selection responses were still achieved. Rasmusson and Phillips (1997) explored this question of continued response to selection in six-row malting barley adapted to the Upper Midwest of North America. Their insights and hypotheses were, alas, not followed up on in a systematic fashion due to the aforementioned curtailment of six-row malting barley production in favor of two-row types.

The recognition that genetic vulnerability and yield plateaus are an inevitable consequence of a narrow germplasm base (Gepts, 2006; McCouch et al., 2013) prompted a systematic search for usable genetic variation in the ancestor of wild barley (*H. vulgare subsp. spontaneum*), land races, and un-adapted germplasm. Recognizing that the distinction between land races and un-adapted germplasm is vague, much of the literature on expanding diversity in locally adapted, cultivated barley is focused on characterization with fewer concrete examples of introgression. While a comprehensive cataloging of germplasm characterization efforts and subsequent introgressions is not within the scope of the current review – the reader is referred to Von Bothmer et al. (2003) we will mention a few illustrative examples. Unique considerations and challenges apply to each of these classes of germplasm – in general it is more difficult to access useful alleles in *H. vulgare subsp. spontaneum*, and not as daunting for unadapted germplasm or land races. These considerations include cross incompatibility, infertility, reduced recombination, and introgression of undesirable alien genome segments resulting in linkage drag.

Starting with *H. vulgare subsp. spontaneum* (hereafter referred to as spontaneum), the potential value of the ancestral species has been well-documented via systematic characterization of phenotypic and genetic variation (Bedada et al., 2014; Sallam et al., 2017); Ongoing efforts to introgress low temperature tolerance alleles from spontaneum are promising (B. Steffenson, personal communication; Lei et al., 2019). Matus et al. (2003) developed a set of recombinant chromosome substitution lines (RCSLs) using a spontaneum donor and an elite cultivar recurrent parent. One of the RCSLs (RCSL-124) advanced to an on-farm trial for commercial assessment prior to release as a variety. Unfortunately, it did not have a yield advantage over the best available feed variety and therefore was as not released (unpublished data). Likewise, spontaneum is the source of novel lipoxygenase (LOX) alleles (Hirota et al., 2008) but the commercially deployed allele was identified in mutants generated in cultivated barley (Skadhauge et al., 2016).

Land races are often described as reservoirs of useful genetic variation for barley improvement and have been used for that purposed (Monteagudo et al., 2019). Historically, land races were a key resource for introgressing alleles into pure line varieties – an example is the *Rpg1* allele tracing to the landrace “Chevron” that subsequently protected North American barley from stem rust (incited by *Puccinia graminus* f.sp. *tritici*) for decades (Steffenson, 1992). In general, however, introgression of favorable alleles from land races into adapted germplasm involves choosing specific exemplars (accessions) for crossing – and this selection can obviate the stated advantages of the land race – which include heterogeneity and potentially heterozygosity (Poets et al., 2015). There is a rich literature on the improvement of land races, particularly in the context of farmer participatory plant breeding, and this was a key emphasis in the ICARDA barley improvement program, formerly based in Syria (Ceccarelli and Grando, 2000; Ceccarelli et al., 2000).

Germplasm collections such as the Leibniz Institute of Plant Genetics and Crop Plant Research (IPK, Germany), the Okayama University Barley and Wild Plant Resource Center (Japan), the International Center for Agricultural research in the Dry Area (ICARDA) and the United States Department of Agriculture National Small Grains Collection (USDA-NSGC, United States) are excellent sources of genetic diversity. The latter contains 29,870 barley accessions including cultivars, breeding lines, land races, wild relatives and genetic stock from more than 100 countries (Bockelman and Valkoun, 2011) and has been extensively characterized for a range of economically important traits (Dahleen et al., 2012; Muñoz-Amatriaín et al., 2014a; Hemshrot et al., 2019). These collections, and others like them, are a rich source of germplasm for finding novel alleles for disease resistance (Czembor, 2000; Yun et al., 2006) drought tolerance (Talamé et al., 2004; Monteagudo et al., 2019) cold tolerance (Visioni et al., 2013; TCAP, 2014) yield (Nice et al., 2019) and other critical traits. One specific example of the effective use of the United States collection is introgressing resistance to the Russian Wheat Aphid (*Diuraphis noxia*). The cultivar “Burton” was developed with RWA resistance contributed by PI 366450 from Afghanistan (Bregitzer et al., 2005). In the case of the Okayama University collection, its accession have served as donors of alleles conferring resistance to Barley Yellow Mosaic Virus (Okada et al., 2004).

One consideration with documenting the effective use of germplasm collections is the time interval between introgression and variety release: pre-breeding can be a lengthy process that is not necessarily amenable to publication in peer reviewed journals. The availability of high-throughput tools, described in the next section, is setting the stage for effective introgression form germplasm collections.

### Current Tools for Assessing and Exploiting Genetic Variation

Next-generation sequencing (NGS) technologies have provided cost-effective methods for surveying genome-wide variation and optimistically will facilitate not only germplasm characterization but also cost-effective and efficient introgression breeding. The use of high density single nucleotide polymorphism (SNP) genotyping platforms (Comadran et al., 2012; Bayer et al., 2017) has provided valuable insights into population structure in barley germplasm arrays that, in turn, generate clues regarding domestication, geographical origin, migration, recombination, and allelic diversity (Muñoz-Amatriaín et al., 2014b; Voss-Fels et al., 2015; Milner et al., 2019). Two of these platforms figure prominently in our own introgression efforts. Data from the re-sequencing of transcriptomes was used to develop the Illumina Infinium 9K assay, in which 7,842 SNPs can be tested simultaneously (Comadran et al., 2012). A more recent genotyping array was constructed based on DNA variant calling using exon capture (EC) in a range of European barley germplasm. This new Illumina Infinium 50K iSelect genotyping array integrates previous genotyping information from the 9K array to provide 43,461 SNPs (6,251 9K + 37,789 EC) that are available for genetic mapping and diversity analysis (Bayer et al., 2017). The sequenced barley genome (Mascher et al., 2017) along with bioinformatic tools, will facilitate the introgression of targeted genomic regions identified during the genetic characterization of diverse germplasm. Examples of this integration of SNP variation with barley genome sequence coordinates are provided in the haplotype visualizations we generated to describe outcomes of our introgression breeding efforts, as detailed in the following sections(Figures 1a, 1b, 1c).

**FIGURE 1a:**
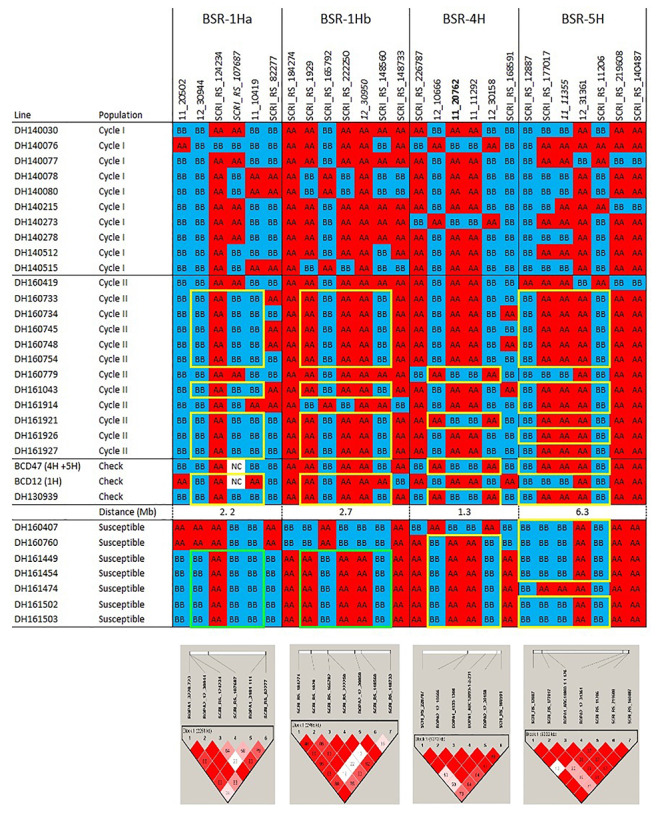
Haplotypes and linkage disequilibrium heat maps based on high density SNP genotyping of Cycle I and Cycle II introgression lines that are resistant to barley stripe rust compared to resistant and susceptible checks. Details on the resistance QTLs are provided in the narrative. The size of each QTL interval (in Mb) is inferred from the barley consensus sequence. Most significant SNPs are in bold. Closer SNP from most significant marker in italic.

**FIGURE 1b:**
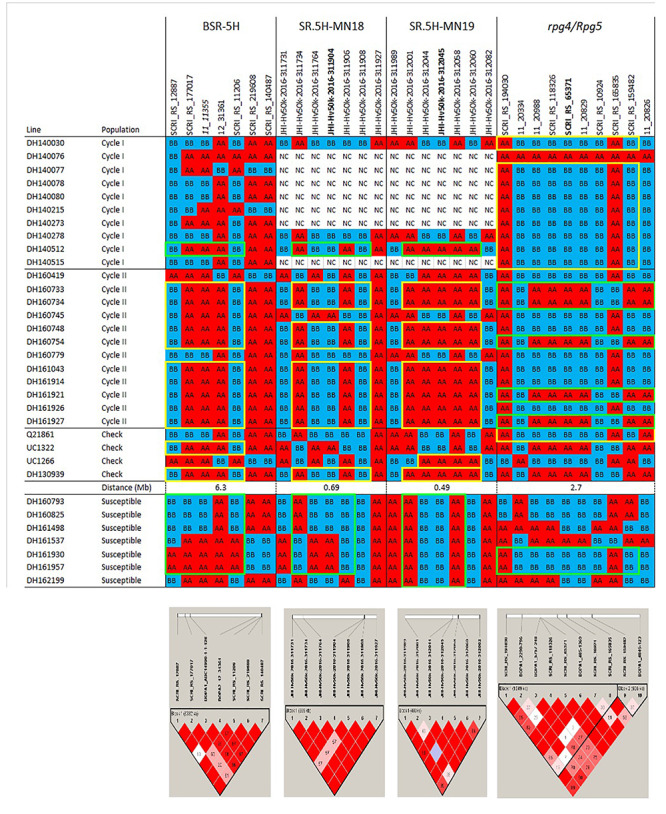
Haplotypes and linkage disequilibrium heat maps based on high density SNP genotyping of Cycle I and Cycle II introgression lines resistant to barley stem rust compared to resistant and susceptible checks. Details on the resistance QTLs and *rpg4/Rpg5* are provided in the narrative. The size of each QTL interval (in Mb) is inferred from the barley consensus sequence. Most significant SNPs are in bold. Closer SNP from most significant marker in italic.

**FIGURE 1c:**
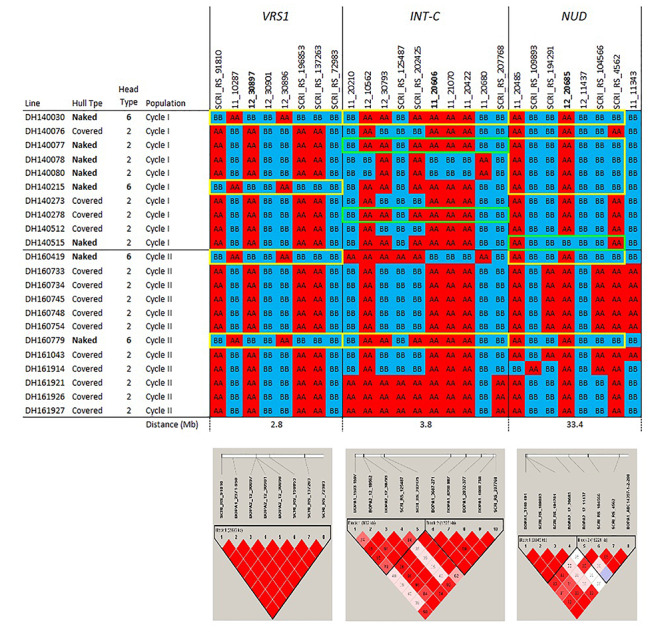
Haplotypes and linkage disequilibrium heat maps based on high density SNP genotyping of selected Cycle I and Cycle II introgression lines at loci determining inflorescence type (*VRS1* and *INT-C*) and hull adherence (*NUD*). Details on the genes determining these morphological traits are provided in the narrative. The size of each introgression interval (in Mb) is inferred from the barley consensus sequence. Most significant SNPs are in bold. Closer SNP from most significant marker in italic.

A drawback to any array is ascertainment bias: the true variants affecting target traits, particularly INDELs, may not be represented in the germplasm used to develop the SNP array (Ganal et al., 2009; Davey et al., 2011). This drawback can be overcome, to some extent, by relying on the linkage disequilibrium (LD) of SNPs that are in LD with causal genes that are underlying the targeted phenotypic differences (Flint-Garcia et al., 2003; Myles et al., 2009; Lipka et al., 2015). Besides this potential downside, the use of NGS methods to rapidly discover thousands of genetic variants in coding or non-coding regions is becoming a standard tool for plant breeders to characterize existing germplasm, analyze genes/QTLs underlying traits of interest, estimate breeding values based on genotypic information, conduct marker assisted selection (MAS) and genomic selection (GS), and target specific alleles in the population (Muñoz-Amatriaín et al., 2014b; Varshney et al., 2014).

Of equal importance to high throughput genotyping are tools for identifying significant marker associations with traits of interest. Bi-parental populations and genome wide association studies (GWAS) are widely used by barley breeders and geneticists to reveal the genetic architecture of simple and complex traits. In barley, there are many examples of crossing two dissimilar parents to dissect simple and complex traits (Castro et al., 2003; Fisk et al., 2013; Esvelt et al., 2016). However, this approach has the constraints of testing just two alleles per locus at a time, low mapping resolution due to limited recombination events, and unrevealed polymorphisms between parents in some genomic regions linked with the trait (Bernardo, 2008; Würschum, 2012). GWAS takes advantage of historical recombination present in an uncontrolled population, which allows for higher resolution mapping (Rafalski, 2010; Bush and Moore, 2012). GWAS has been used in barley for characterizing the genetic basis of traits including growth habit, disease resistance, phenology, and end-use quality (Cuesta-Marcos et al., 2010; Visioni et al., 2013; Muñoz-Amatriaín et al., 2014a; Graebner et al., 2015; Gutiérrez et al., 2015), disease resistance (Sallam et al., 2017; Case et al., 2018), and drought and salt tolerance (Thabet et al., 2018; Xue et al., 2019). Quantitative trait loci (QTL) identified through either or both of these methods can then be targeted via (MAS) and/or used to monitor the effects of GS.

The identification of QTL through the integration of genotypic and phenotypic information sets the stage for introgression. In principle, this is as straightforward as using QTL as a platform for MAS (Babu et al., 2004; St Clair, 2010). Based on this approach, wild type barley accessions are a rich source of favorable alleles for yield, malting quality and disease resistance in barley. As an example, lines derived from *Hordeum bulbosum*, a secondary barley gene pool, have been used to characterize resistance and agronomic relevant traits (Pickering et al., 2004; Johnston et al., 2013; Czembor et al., 2019; Hoseinzadeh et al., 2020). Accessions derived from *spontaneum* are also a source of novel and potentially useful alleles (Matus et al., 2003; von Korff et al., 2008; Nice et al., 2019). The dilemma is that as the donors get more exotic, the more likely it is that there will be linkage drag. Matus et al. (2003) found that the *spontaneum* accession Caesarea 26–24 had a negative effect on the variety Harrington in terms of agronomic performance and malting quality. For this reason, during the introgression process, it is important to reduce the size of the chromosome section carrying the targeted genomic region. This is important because potentially undesirable genes with negative effects on important traits may be physically linked with the target donor allele(s) (Hospital, 2005). Without the use of markers defining QTL regions, these donor segments can be quite large, which increases the chance of undesirable genes ending up in the recurrent genetic background (Ribaut et al., 2002; Salina et al., 2003). One example of the application of markers to reduce linkage drag is the advanced back-cross method. This approach has been used in barley for several traits including disease resistance, malting quality, and yield (Matus et al., 2003; von Korff et al., 2008). High-resolution genotyping technologies can assist in overcoming the problem of linkage drag by providing better map/sequence resolution of the target allele(s) and, as a result, a reduction in the size of the introgressed DNA segments. However, even when the size of the introgression segments can be successfully reduced, the favorable alleles from the exotic germplasm may not have predictable phenotypic effects in new genetic backgrounds (Richardson et al., 2006). Therefore, a validation process of assessing novel qualitative, or quantitative, trait alleles is warranted (Bilgic et al., 2005; Richardson et al., 2006; Sharma et al., 2018; Hernandez et al., 2019).

## A Case Study in Characterization and Introgression: Multi-Rust Resistance

Stripe rust (incited by *Puccinia striiformis* f. sp. *hordei*) and stem rust (incited by *Puccinia graminis* f. sp. *tritici*) are barley diseases of worldwide importance. Stripe rust resistance has long been a focus of our program, due to its prevalence in the Pacific Northwest of the United States. Briefly, barley stripe rust (BSR) was first reported in the Americas in 1976, when it was discovered in Colombia (Dubin and Stubbs, 1986). The disease spread throughout the Americas, arriving in the United States in 1991. A long-term collaboration with the late Dr. Hugo Vivar, who led the former ICARDA barley program based at CIMMYT in Mexico, resulted in extensive literature on mapping resistance genes and QTLs (most recently reviewed by Belcher et al., 2018). Parallel to these efforts we conducted ongoing stripe resistance breeding efforts based primarily on phenotypic selection because (1) mapping efforts were conducted in spring growth habit barley and our breeding program is directed primarily at winter and facultative growth habit barley and (2) phenotypic selection is generally effective at our test sites due to high heritability and consistent natural disease epidemics. Periodically, however, QTL alleles were characterized in germplasm derived from phenotypic selection in our winter and facultative barley program (Belcher et al., 2018). We added stem rust to our resistance breeding efforts due to the threat posed by race TTKSK of this disease, which has yet to be reported in the Americas. Breeding for resistance to these two rusts has allowed us to integrate characterization, validation, and introgression within a coordinated framework. A discussion of the framework of targeted introgression of resistance to race TTKSK, stripe rust, and, to a limited extent leaf rust now follows (Hernandez et al., 2020).

### Cycle I Population

The resistance gene *Rpg1* has been used as the primary source of stem rust resistance since a major epidemic occurred in the United States in the 1940s (Steffenson, 1992). As noted earlier in this chapter, this gene was introgressed from a land race into the principal barley cultivars grown in the upper Midwest of the United States and the Canadian Prairies. During the late 1980s, *Rpg1* was defeated by race QCCJB, demonstrating the urgency of finding new sources of resistance to stem rust (Jin et al., 1994). The urgency became acute with race TTKSK, which is virulent to *Rpg1*: 95% of commercial barley cultivars are susceptible to this race (Steffenson et al., 2017). Fortunately, this race is yet to be reported in the Americas, which provides an opportunity to engage in defensive resistance introgression breeding. An accession – Q21861 – carries the resistance complex *rpg4/Rpg5* which confers resistance to several races of stem rust including QCCJB and TTKSK at all growth stages (Steffenson et al., 2009). This accession was developed by same Dr. Hugo Vivar with whom we had collaborated on stripe rust resistance. This unique and valuable accession can be considered an un-adapted elite line: it does not have the requisite productivity and end-use quality for direct release as a variety in North America. It did, however, provide a starting point for multiple programs to work defensively, via introgression, to prepare for the inevitable arrival of race TTKSK.

**FIGURE 2:**
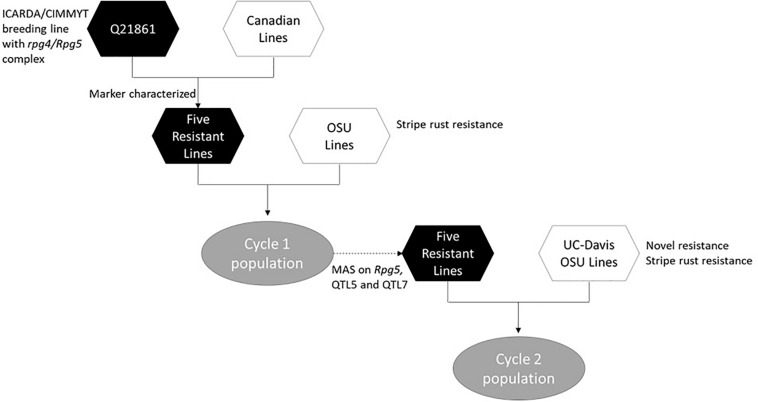
Flow chart showing the introgression process involved in development of the Cycle I and Cycle II barley populations.

The process of introgression of the *rpg4/Rpg5* complex in our program is shown in Figure 2. As described by Hernandez et al. (2019), germplasm was obtained from Canadian programs that, in turn, was derived from introgression of *rpg4/Rpg5* from Q21861. This Canadian “pre-breeding” germplasm (five accessions) was used in crosses with varieties and elite germplasm from our own and other breeding programs (both covered and naked) that was resistant to stripe rust. In addition, we used as parents two accessions of Himalayan origin – one from the USDA-GRIN collection and one from the James Hutton Institute (Scotland) collection, and a land race from Washington, United States. One hundred and nineteen doubled haploid (DH) lines were generated from these F1s: this array is referred to as “Cycle I” – the first step in introgressing resistance to TTKSK into adapted germplasm. Using an allele-specific marker for *Rpg5* and SNP data from the Illumina 9K platform, we showed that in the Cycle I population the *rpg4/Rpg5* complex is required but not sufficient to confer stem rust resistance at the seedling stage in a diverse array of genetic backgrounds. Using GWAS, two other loci – one on chromosome 5H and one on chromosome 7H – were found to be associated with resistance and interacting with the *rpg4/Rpg5* complex. Sharma et al. (2018) also reported that an additional gene – in this case denominated *Rrr1* (required for *rpg4*-mediated resistance 1) – is required for the *rpg4/Rpg5* complex to confer resistance when introgressed into the variety “Pinnacle.” The Cycle I germplasm was also phenotyped for adult plant resistance to stripe rust and leaf rust (incited by *Puccinia hordei*). Due to the limited number of environments where these diseases occurred, results were not included in the Hernandez et al. (2019) paper. In the context of this chapter, it is worth reporting the GWAS results and integrating them into the stem/stripe rust introgression story. Complete data are available at Barley World^1^. The combined GWAS of all available data (2 years, two locations) identified a significant QTL associated with stripe rust resistance on chromosome 5H at the same position as the adult plant resistance stripe rust QTL we found in Cycle II and reported in Hernandez et al. (2020). Based on one year/location of data, a QTL was identified on chromosome 7H, coincident with a leaf rust QTL reported by Gutiérrez et al. (2015) that traces back to ICARDA/CIMMYT/Mexico germplasm. Ten Cycle I doubled haploids were identified with resistance to all three rusts (Table 1). Of these ten, five (highlighted) were selected as parents for a second round of introgression and validation (Cycle II).

**TABLE 1:** Selected doubled haploids from the Cycle I and Cycle II populations with resistance to stripe rust (incited by *Puccinia striiformis* f. sp. *hordei*), leaf rust (incited by *Puccinia hordei*) and stem rust (incited by *Puccinia graminis* f. sp. *tritici*).

Line	Pedigree	Population	GHF	FHD	IT-M (SR)^a^	Sev (SR)^b^	Sev (BSR)^c^	LR^d^	Head type	Hull type
**DH140278**^e^	SH98076/Full Pint	Cycle I	52	137.5	0;1-	10	10	13.7	Two	Covered
DH140078	SH98076/10.1151	Cycle I	75	139.5	1,0;	ND	10	3	Two	Naked
**DH140512**	SH98076/Full Pint	Cycle I	65	138.5	2,1	1	6	8.7	Two	Covered
DH140080	SH98076/10.1151	Cycle I	61	136	2,1,0;	10	1.7	7.3	Two	Naked
DH140515	SH98076/10.1151	Cycle I	68	138.5	1,0;	1	3.3	7.3	Two	Naked
**DH140030**	SH98076/10.1151	Cycle I	100	133	1,0;,2	10	11.7	10	Six	Naked
DH140077	Violetta/SH98076	Cycle I	104	137.5	2,1	1	3.3	10.3	Two	Naked
DH140076	MC0181-11/Full Pint	Cycle I	59	138.5	2,1	1	8.3	3.3	Two	Covered
DH140273	SH98076/Full Pint	Cycle I	50	133.5	1,2	10	3.3	10.3	Two	Covered
DH140215	SH98076/10.1151	Cycle I	42	130.5	1,2,0;	ND	3.3	10.7	Six	Naked
DH160419	UC1266/DH140213	Cycle II	63	121	0;1	7.25	6.4	ND	Six	Naked
DH160733	DH140512/UC1322	Cycle II	66	110	0;1-	5	2.5	ND	Two	Covered
DH160734	DH140512/UC1322	Cycle II	64	109.5	0;	10.75	2.5	ND	Two	Covered
DH160745	DH140512/UC1322	Cycle II	70	108.5	0;	8.25	2.6	ND	Two	Covered
DH160748	DH140512/UC1322	Cycle II	66	112	0;	2.5	2.6	ND	Two	Covered
DH160754	DH140512/UC1322	Cycle II	68	111	0;	4	2	ND	Two	Covered
DH161043	DH140512/UC1322	Cycle II	64	110.5	0;1	3	2.5	ND	Two	Covered
DH161921	DH140512/DH130004	Cycle II	55	122	0;1	11.75	3.5	ND	Two	Covered
DH161926	DH140512/DH130004	Cycle II	65	114	0;1	5	8.1	ND	Two	Covered
DH161927	DH140512/DH130004	Cycle II	65	117.5	0;1	13.75	7.5	ND	Two	Covered
DH161914	DH140512/10.0860	Cycle II	64	113.5	0;1-	6	3.1	ND	Two	Covered
DH160779	DH140030/UC1231L	Cycle II	62	115.5	10;	3.5	4.2	ND	Six	Naked

*^*a*^Infection-type mode (IT-M) after infection by *Puccinia graminis* f. sp. *tritici* race TTKSK at seedling stage. Data was collected in the Biosafety Level 3 Containment Facility at University of Minnesota. ^*b*^Disease severity of stem rust expressed as percentage at adult stage. For Cycle I, data collected under field evaluations in Kenia during 2017. For Cycle II, line means of data collected in field evaluations in Saint Paul, Minnesota during 2018 and 2019 seasons. ^*c*^Disease severity of stripe rust expressed as percentage at adult stage. For Cycle I, data collected under field evaluations during 2017. For Cycle II, values represent the best linear unbiased predictor (BLUPs) for stripe rust severity after infection by *Puccinia striiformis* f. sp. *hordei*, across 2018-2019 seasons in Davis, CA and Corvallis, OR. ^*d*^Disease severity of leaf rust expressed as percentage at adult stage. Data collected under field evaluations during 2017. ^*e*^Lines in bold were used as parents for Cycle II population.*

### Cycle II Population

The Cycle II population is comprized of 358 doubled haploids derived from crosses of the five selected Cycle I lines with five elite lines from our program and three elite, un-adapted lines from the University of California – Davis barley breeding program (Hernandez et al., 2020). The goal of Cycle II was to continue introgression of multi-rust resistance alleles into elite germplasm. In Cycle II we assessed seedling resistance to TTKSK as we did with Cycle I, under tightly controlled environmental conditions, and added assessment of adult plant resistance under field conditions using race QCCJ as a surrogate for race TTKSK. We also assessed adult plant resistance to stripe rust, as we did with Cycle I. Leaf rust epidemics were not sufficiently severe in any of the field trials to generate data for QTL analysis. Reinforcing the importance of phenotyping introgression lines as extensively as possible, using GWAS we found different genes and/or QTL related to resistance to stem rust at the seedling and adult plant stages. While *rpg4/Rpg5* was a principal determinant of resistance at the seedling stage – it was not effective at the adult plant stage in one year. We hypothesized that the difference in resistance at the adult plant stage was due to temperature differences in the two years of testing: it is known that the *rpg4/Rpg5* complex does not confer resistance under high temperature conditions. A QTL on 5H, mapping to a different position than that identified in Cycle I, was associated with adult plant resistance under high temperature, and it is coincident with one of the three QTLs conferring resistance to stripe rust at the adult plant stage. Other significant stripe rust QTLs were identified on 1H and 4H. The 1H QTL is coincident with that reported by Toojinda et al. (2000), which traces to the variety Shyri, released in Ecuador by the ICARDA/CIMMYT program. Subsequently, Castro et al. (2002a) and Richardson et al. (2006) introgressed this allele into susceptible elite backgrounds and validated its effectiveness. The 4H QTL is also coincident with prior reports and traces to Calicuchima-sib, also from the ICARDA/CIMMYT program (Chen et al., 1994). This 4H allele was subsequently validated in other elite genetic backgrounds after further cycles of introgression (Castro et al., 2002b) and was independently identified in unrelated germplasm by Esvelt et al. (2016). Calicuchima-sib was also the donor of the 5H QTL (Chen et al., 1994; Castro et al., 2002b). We have selected 12 doubled haploids from Cycle II with resistance to both stem and stripe rust. In summary, the introgression of multiple alleles from different regions of the genome was successful in conferring resistance to stripe, stem, and leaf rust. In the case of stripe rust and stem rust, the introgressed resistance alleles trace to the ICARDA/CIMMYT program based in Mexico – testimony to the effectiveness of this program in pyramiding resistance based on phenotype alone. Further research is needed to validate the leaf rust resistance QTL allele.

The pedigrees and phenotypes of the 22 doubled haploids selected from Cycles I and II are provided in Table 1 and this germplasm is freely available for research purposes. Haplotype analysis provides insights into the genetic architecture of these introgression lines and addresses key issues in introgression breeding, such as the discriminatory power of marker haplotype information, extent of LD, and how these alleles interact with the genetic background they are introgressed into. The most phenotypically resistant lines from Cycle I and Cycle II were used to identify haplotypes associated with biotic and morphological traits based on high throughput genotyping arrays (Figures 1a, 1b, 1c). In the case of stripe rust resistance, a defined pattern is observed in lines carrying the resistance haplotype on 5H and 4H. These same haplotypes are observed in the resistant check BCD47 (Castro et al., 2003) and DH130939, a facultative breeding line with phenotypic resistance. For the QTLs on 1H, the haplotype is not as predictive. Q21861 is a well-known source of stem rust resistance carrying both *Rpg1* and the *rpg4/Rpg5* complex. This resistance was the foundation for mapping and introgression of stem rust resistance into the more adapted germplasm generated from the Cycle I and Cycle II populations. Based on the seedling stage resistance phenotype, there is a clear *rpg4/Rpg5* diagnostic haplotype for resistant lines in Cycle I and the Q21861 check. A similar pattern is observed in Cycle II, were half of the TTKSK-resistant lines have the same haplotype. Interestingly, the other half of the Cycle II resistant lines with seedling resistance to TTKSK and adult plant resistance to the surrogate race (QCCJ-B) have a distinct haplotype compared to Q21861. This haplotype is observed in the donors of a potentially new source of resistance (UC1322, UC1266 and, DH13939) and these donors share a haplotype in common at the adult plant resistance QTL on 5H. This 5H QTL for adult plant resistance to stem rust is coincident with the adult plant resistance QTL for stripe rust. For inflorescence type, at *VRS1*, all six-rows have a distinctive haplotype as compared to two-rows. At the *Int-C* locus (which determines the size of lateral florets), there is no defined haplotype. At the *Nud* locus, seven out eight naked lines share the same haplotype. The one exception merits further research. The LD among markers close to target loci was evaluated to identify if haplotype structures were constant across lines. In general, LD was high between the markers across all the loci evaluated for disease resistance and morphological traits. In a few cases (e.g., *Nud, INT-C* and *rpg4/Rpg5*), two blocks were identified among markers.

## Next Steps in Introgression Breeding: End-Use Quality

Currently, there is fragmentation in barley production for different end-uses due to the naked vs. covered grain phenotype, the genetic basis of which was described earlier in this chapter. We are initiating a collaborative effort to develop naked barley germplasm that will have sufficient quality and productivity to be used for food, malt, and/or feed. This effort is based on our recent review on the topic of breeding naked barley for multiple end-uses (Meints and Hayes, 2020) and will make use of NGS technologies. Faced with a lack of adapted naked barley germplasm resources, we have developed a plan for systematic introgression of target alleles, early generation marker-assisted selection, speed breeding, and SNP genotyping. The program is now at the implementation stage, and the general framework is a modified Nested Association Mapping (NAM) population that will allow for simultaneous introgression, development of enhanced germplasm and potential varieties, and gene discovery.

**FIGURE 3:**
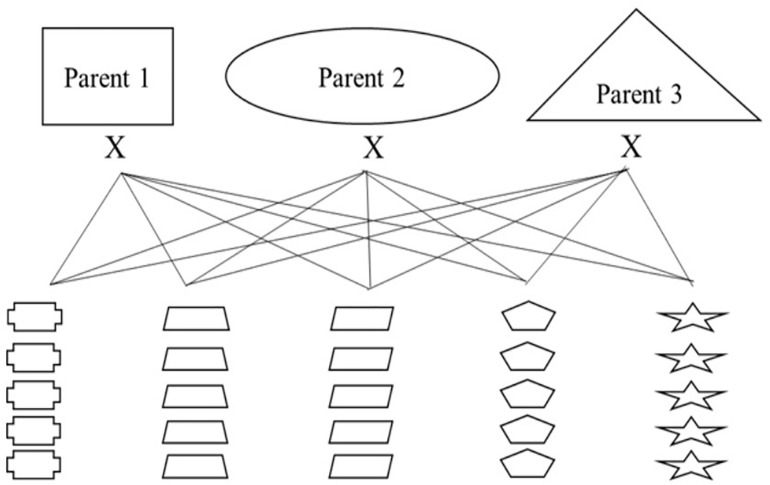
An introgression breeding scheme for modified NAM population development using naked, multi-use barley for organic systems as a model.

Nested association mapping (NAM) populations are multi-parent panels that are designed to combine the advantages of linkage analysis and association mapping in order to delve into genomic regions of interest (Maurer et al., 2015; Hemshrot et al., 2019). Additionally, these panels can be designed as breeding populations to select for new varieties with traits of interest. In order to breed for multi-use naked barley and explore regions of the genome associated with quality traits and the agronomic performance of naked barley, our modified NAM population will have three common parents each crossed to 25 regional parents selected by cooperating breeding programs, for a total of 75 crosses (see Figure 3). The three common parents are elite naked breeding lines and the 25 regional parents are a combination of un-adapted breeding lines and land races from USDA-GRIN chosen because they contain target alleles for traits of interest that will be introgressed into the elite parents. Thus, every cross will either be segregating for the naked grain trait or will be fixed for the trait. The overall breeding targets for the entire population are based on traits that are important for multi-use barley and include: naked caryopsis, facultative growth habit, two-row inflorescence, good threshability, and modest β-glucan.

In order to conserve space and select for highly heritable desired traits as soon as possible, a panel of high-throughput allele-specific markers will be used for MAS at the F_2_ stage. The target loci are described in Table 2. All lines will be selected for the *nud* allele and a combination of the other alleles based on the traits targeted in that specific cross. By selecting for desired phenotypes at the F_2_ stage, some genetic variation will be removed from the population, resulting in a modified NAM population rather than a true one. However, the population will still be useful for later GWAS. A potential drawback of using MAS at the F_2_ stage to fix desirable alleles is that this will also reduce recombination in these regions and will create linkage blocks around the MAS targets. This will be problematic if undesirable alleles are linked to the favorable target alleles. To mitigate this potential impact, heterozygotes will be selected at the F_2_ stage for target regions in a subset of crosses and advance them by single seed decent (SSD) imposing MAS for the heterozygote for several generations. After selfing the heterozygote, near-isogenic lines will be recovered for each target region in a selection of genetic backgrounds that can be used to (i) validate and quantify the value of the targeted loci; (ii) determine if there are undesirable traits linked to the targeted allele; (iii) develop a set of near isogenic parents that can be used to fine map the region and recover recombinants that resolve unfavorable linkages.

**TABLE 2:** Genes/QTLs targeted for marker assisted selection in the modified NAM population shown in Figure 3.

Targeted allele	Selection target	Citation
*nud*	Naked caryopsis	Taketa et al. (2008)
*Vrs1*	2-row spike	Komatsuda et al. (2007)
*Wx*	Normal starch	Patron et al. (2002)
Deletion at *Vrn-H2*	Facultative	Karsai et al. (2005)
*Ppd-H2*	Short photoperiod sensitivity	Laurie et al. (1995)
*Rpg1*	Stem rust resistance	Brueggeman et al. (2002)
*Run8*	Resistance to loose smut	Grewal et al. (2008)
*Ruhq and Ruh1*	Resistance to covered smut	Grewal et al. (2008)
*Three locus haplotype*	Resistance to spot blotch	Haas et al. (2016)

The modified NAM population will to be advanced through SSD using “speed breeding,” a method in which increased daylight hours and temperatures decrease generation time (Heuschele et al., 2019). The population will be advanced in the greenhouse through at least the F_5_ generation to increase homogeneity before field testing, where the lines will be assessed for agronomic performance, resistance to biotic and abiotic stresses, and end-use quality traits. This breeding scheme will allow for introgression of resistance to biotic and abiotic stresses and quality and agronomic traits into elite naked barley germplasm that will result in potential new multi-use cultivars and germplasm resources for other breeding programs.

## Conclusion and General Perspectives

Introgression breeding has been, remains, and will be a feature of barley improvement – providing an essential tool to meet the challenges of climate change, ensuring profitable and sustainable production, and enhancing both nutrition and flavor. The donors of alleles for introgression are, not surprisingly, more frequently reported in elite and/or “pre-bred” un-adapted germplasm, followed by land races and exotic accessions in germplasm collections, and finally by wild relatives. Breeders are, not surprisingly, loathe to range far afield in the gene pool, because this increases the risk of linkage drag and/or disruption of the carefully constructed genome architectures that determine adaptation, meet productivity expectations, and ensure end-use quality. Someone, somewhere, however, needs to assume the risk of conducting the essential pre-breeding required to introgress alleles from the wild and exotic into more adapted backgrounds. An example of the effectiveness of such efforts was the ICARDA/CIMMYT barley program based in Mexico.

The availability of cost-effective, high throughput genotyping tools and analysis procedures has facilitated a plethora of germplasm characterization and allele-discovery studies. Effective mining of these alleles will optimistically follow, as the same tools can be used to track and validate the effects of introgression of these novel alleles into adapted germplasm. However, an interesting alternative emerges as QTLs are reduced to candidate genes and the costs of whole genome and targeted allele sequencing decline to the point of pricing them within reach of breeding programs.

The alternative is gene editing, as currently implemented by CRISPR-Cas9. If the target allele sequence is known - and its function understood - in un-adapted germplasm, a germplasm collection accession, or a wild relative, it is conceptually appealing to sidestep the Scylla and Charybdis of linkage drag and disruption of genome architecture by “simply” knocking-out/knocking-in the allele in adapted germplasm. “Simply” appears in quotes in the preceding sentence because although the gene editing process appears straightforward, it in fact requires a comprehensive understanding of gene function in order to know what to edit and how. Furthermore, germplasm specificity can limit what genotypes can be edited: this leads to the reliance on one or a few “workhorse” genotypes amenable to the transformation processes that can precede editing. In the cased of barley, this would mean that significant efforts would be required to introgress edited alleles from the highly transformable variety “Golden Promise” into target adapted backgrounds. At that point, breeders might question the merits of genome editing – which will likely involve regulatory hurdles and/or intellectual property costs – and instead choose to engage in the time-honored processes of crossing and selection.

## Author Contributions

JH and PH provided the outlines of the review and key concepts. JH wrote a draft layout of the manuscript. JH, BM, and PH contributed to the writing, editing and final draft of this review. All authors read and approved the manuscript.

## Conflict of Interest

The authors declare that the research was conducted in the absence of any commercial or financial relationships that could be construed as a potential conflict of interest.

## Footnotes

1 https://barleyworld.org/barley-stripe-rust-bsr
